# A Digital Cueing Intervention for Parkinsonian Gait: Laboratory-Based Clinical Validation and Acute Gait Responses

**DOI:** 10.1007/s10916-026-02441-x

**Published:** 2026-07-11

**Authors:** Conor Wall, Victoria Hetherington, Rodrigo Vitorio, Peter McMeekin, Richard Walker, Jason Moore, Rosie Morris, Yunus Celik, Alan Godfrey

**Affiliations:** 1 School of Computer Science, Northumbria University, Newcastle upon Tyne, NE1 8ST UK; 2 Cumbria Northumberland Tyne and Wear NHS Foundation Trust, Newcastle upon Tyne, UK; 3 School of Sport, Exercise and Rehabilitation, Northumbria University, Newcastle upon Tyne, UK; 4 School of Healthcare and Nursing Sciences, Northumbria University, Newcastle Upon Tyne, UK; 5 Northumbria Healthcare NHS Foundation Trust, North Shields, UK; 6 Department of Electrical and Electronics Engineering, Istanbul University-Cerrahpasa, Istanbul, Turkey

**Keywords:** Parkinson’s disease, Personalised cueing, Smartphone, Fall risk, Clinical validation

## Abstract

Falls are common in people with Parkinson’s (PwP). There is a need for a scalable, pragmatic, personalisable, and single device intervention to improve gait characteristics related to fall risk. CuePD is an app-only approach to deliver personalised auditory cues to retrain gait in PwP. This study aimed to clinically validate and evaluate effectiveness of CuePD in PwP and to explore their perceptions of CuePD. Sixty/60 PwP performed cued walks via metronome, instrumental and vocal music personalised to their baseline walking speed (cadence). PwP chose music from e.g., pop, rock genres. Cued walks were +10% on baseline cadence. We examined effectiveness of each cue and any carryover effects across non-cued walks. CuePD clinical validation (e.g., intraclass correlation coefficients, ICC_(2,1)_) was established against a reference standard. CuePD effectiveness was examined via improvement (e.g., standardised response means) of gait characteristics indicative of fall risk (stride length, walking speed, step time coefficient of variation). CuePD quantified fall relevant mean gait characteristics against a reference system with good-to-excellent agreement (ICC_(2,1)_ 0.849 – 0.986), though agreement was poor for several variability and asymmetry measures. Vocal music was the most preferred cue type, improving gait characteristics by increasing stride length (7cm, SRM of 0.873) and gait speed (0.115 m/s, SRM of 1.264), while reducing stride time coefficient of variation (-0.418%, SRM of-0.505). CuePD received positive feedback and is an app-only approach that is clinically valid to quantify mean gait characteristics and has demonstrated acute improvements in gait characteristics related to fall risk. Findings suggest that the personalised approach may have pragmatic utility beyond the lab, but ecological validity needs to be established.

**Supplementary Information:**

The online version contains supplementary material available at 10.1007/s10916-026-02441-x.

## Introduction

Falls and recurrent falls [1] are a significant and disabling problem for many people with Parkinson’s (PwP) [2, 3]. A recent patient-and-family priority study [4] found that PwP identify movement-related symptoms (e.g., gait) as most bothersome, while families prioritise physical and psychosocial impacts such as safety, independence, and family impact. As gait is fundamental to everyday functioning and independence, mechanisms to improve gait via targeted intervention could markedly improve safety, quality of life, and reduce burden of care [5]. Digital approaches e.g., inertial sensors (accelerometers, gyroscopes), are long proposed as viable methods for (i) improved fall prediction models via the implementation of movement assessment tools and/or (ii) closed-loop cueing and feedback technologies for use in the home [6–9].

Inertial sensors are a near ubiquitous technology in contemporary life and are commonly embedded within e.g., smartphones and wearables. Therefore, their use for assessing mobility, motor performance, and movement disorders has risen sharply [8, 10]. The Movement Disorders Society (MDS) Task Force on Technology defined that there is a need to develop and concurrently integrate mobile health technologies into (i) the routine assessment and (ii) care of PwP [11]. Importantly, the MDS Task Force suggest that a mobile approach needs to be homogenous to (i) target deficits confirmed to be relevant to PwP (ii) be derived from a single device (ideally), that delivers an acceptable benefit-to-burden ratio to patients while yielding clinically useful information and (iii) deliver individualised data to patients (caregivers/care-partners, and clinicians) [11].

Smartphones are now sufficiently mature to support the collection of patient-relevant data [10, 11], with their sensing (inc. inertial sensors) and computing power reporting equal analytical capabilities compared to routine lab-based technologies [11, 12]. As familiar and widely distributed devices, their sophistication may provide a homogenous platform to break remote assessment barriers. Specifically, advanced processing power and internet-of-things functionality could provide prompt outcomes and seamless feedback to PwP and clinicians. Together, these features suggest smartphones may provide a practical and optimal single device, through suitable applications/apps, for providing a well-rounded, holistic tool to reduce fall risk in PwP [12]. Moreover, smartphones provide a ubiquitous technology to enable the capture of clinically relevant gait characteristics indicative of fall risk. Specifically, cadence, stride length, gait speed and coefficient of variation (CV_stride−time_) are established, cue-modifiable markers of mobility and fall risk in PwP [12, 13].

Here, we present CuePD, a novel gait retraining/rehabilitation smartphone app to retrain gait and improve characteristics related to fall risk in PwP. While metronome (ME) cueing is the most established approach in PD [14], its monotony can limit engagement [15]. CuePD aims to offer a more engaging, personalised alternative. Unlike prior approaches that use a limited music selection (i.e., tracks and/or genres) [16] or use of music overlaid with a metronome [17], CuePD enables self-selected, tempo-personalised music, leveraging familiarity and preference which are known to influence cueing response [14]. It does so from a smartphone-based app only to provide both gait assessment and cued intervention without peripheral wearables required by other systems e.g., [18]. The present study forms the clinical validation of CuePD within the V3 framework [19]: verification (V1), analytical validation (V2), and clinical validation (V3). Previously V1 [20] and V2 [21] of CuePD provide the foundation for this current study. Accordingly, this study aimed to (i) evaluate the clinical validity (V3) of CuePD for measuring gait characteristics in PwP against a reference standard, and (ii) assess the acute responsiveness of meaningful gait characteristics often used in fall-risk prediction models to personalised cueing. We also included a battery of assessments to understand PwP use of the app and to inform the next stage of CuePD as a pragmatic, scalable, and personalised gait intervention.

## Methods

This study was performed in line with the principles of the Declaration of Helsinki. Ethics approval was granted by Northumbria University research ethics committee (Ref: 3231) and an NHS research ethics committee (24/PR/0684 via the Integrated Research Application System, #327241) with a Health Research Authority approval letter, 25-Jul-2024. This trial was listed on the ClinicalTrials.gov (NCT06941779: https://clinicaltrials.gov/study/NCT06941779) and the protocol is published [22] but briefly:

- Participants gave informed written consent prior to participation.
- Participants were recruited via the Jubilee Day Hospital (North Tyneside General Hospital, North Shields) and local DeNDRoN research delivery register between 21-11-2024 and 05-03-2026.
- Inclusion criteria included: age > = 50 years, walk unaided, diagnosis of idiopathic PD, and a Montreal Cognitive Assessment (MoCA) score of > = 21.
- Exclusion criteria: see [22].
- Upon attending the gait lab at Northumbria University, participants wore a smartphone on their lower back (at the fifth lumbar vertebrae, L5) via belt-mounted holder with screen facing outward, reference wearables (Opals, APDM, Portland, USA; MobilityLab v2) on dorsal aspect of both feet, and headphones over ears. PwP selected their favourite song from an extensive list spanning popular genres; tempo was personalised via a time-shifting algorithm and volume set by the participant.
- Participants performed a baseline walk at their natural gait speed/cadence and 3 subsequent walks with a cue: metronome (ME), instrumental music (IM), and the same instrumental music with vocals retained (VM). See supplementary material., Figure S1.
- All walks were 1-minute/min and were counterbalanced deterministically i.e., participants were assigned to walk order depending upon their sequence of enrolment, which was not known by participants (see [22]). The cued walks were set at + 10% of baseline cadence, which is shown to ameliorate Parkinson’s gait [23].
- Wash out. Between cued walks, participants (i) sat and counted backwards in their heads from 30 − 0 in increments of 1 to disengage psychological responses triggered by cueing [24] and then (ii) performed a 1-min walk at a natural pace with no cue, to further reset their gait but also to assess whether any carryover effect remained before the next cue. Un-cued washout walks are denoted WO3 and WO5, with WO3 following first cued walk and WO5 after the second cued walk.
- All walks were performed in the same manner around a 25 m track (two curved, two straight sections), supervised by two researchers, walking alongside participants where needed for safety.
- Gait characteristics synonymous of mobility related falls in Parkinson’s [9, 25, 26]: cadence, stride time coefficient of variation (CV_stride-time_), stride length, gait speed.
- A description/functionality of CuePD is provided elsewhere but uses validated gait algorithms for use in PwP [21]. Developed in React Native (cross-platform iOS/Android), tested on iPhone XS/iOS CuePD sampling tri-axial acceleration (100 Hz) and synchronised with reference (128 Hz). A 4th-order 20 Hz Butterworth filter and continuous wavelet transform identified initial/final contacts [27]. All data are processed by CuePD at the end of walks.
- Participants complete the System Usability Scale (SUS) [28], Goldsmiths Musical Sophistication Index (Gold-MSI) [29], and answered structured questions about study experience. Responses were collated as exploratory participant feedback.

### Statistical Analysis

An extensive description is provided in the protocol [22]. In brief, descriptive statistics (e.g., mean, min) and standardised response mean (SRM) summarise changes in gait characteristics, with SRM assessing responsiveness (≥ 0.8 large, 0.5–0.8 moderate, < 0.5 low: where positive values imply an improvement for cadence, gait speed and stride length but negative is an improvement for CV_stride−time_ i.e., reduces variability). Agreement between CuePD and reference are evaluated using ICC_(2,1)_, and linear relationships using Pearson’s correlation coefficient (PCC) (not as evidence of agreement). Agreement is further assessed at participant level using Bland-Altman plots [30], reporting mean bias and 95% limits of agreement (LoA). Cueing adherence is quantified as percentage change in cadence from baseline, with effectiveness defined as change closest to + 10% [23]. Assumptions (normality, variance, sphericity, outliers) are tested using standard methods (Shapiro–Wilk, Levene’s, Mauchly’s). A two-way mixed ANOVA examines effects of cueing modality, walk order, and interaction on gait characteristics, with aligned rank transform (ART) as a nonparametric check. Design was balanced with no missing data, with cue type as a within-subject factor and walk order as a between-subject factor. Outliers are retained, as ART results were consistent throughout. Changes from baseline are expressed as percentage change for gait speed and stride length, and log-ratio change for CV_stride−time_ to address skewness. Post hoc pairwise comparisons (with Bonferroni correction) follow significant main effects, with effect sizes reported. Carryover effects are assessed by comparing changes across sequential walks. Associations between musical sophistication and both cue adherence and gait improvements are evaluated using Spearman’s correlations with correction for multiple comparisons. A sensitivity power analysis indicated with *n* = 60 and 80% power, the Gold-MSI correlations could detect |r|≥0.32; therefore, weak associations could not be reliably detected [31, 32]. Sequence awareness could not influence any outcomes as computed by an automated algorithm.

## Results

Sixty PwP were recruited and all performed each gait task, with no sensor failure/unusable data. No falls or adverse events occurred. Table 1 presents demographic and clinical characteristics. For PwP, 65% (39/60) had not reported a fall in the previous 12-months and many (77%, 46/60) were H&Y stage II. 58/60 participants were in the ON (medicated) state and 2 were unmedicated. None who reported history of FOG (8/60 via UPDRS) was observed to experience FOG during the study.

**Table 1:** Participant clinical and demographic characteristics

Characteristic	Count, mean ± SD
N participants	60
Age (years)	68.2 ± 7.0
Gender (M/F)	38/22
Height (cm)	170.4 ± 9.8
Weight (kg)	79.2 ± 18.0
Years diagnosed	4.8 ± 3.7
UPDRS III	36.9 ± 15.5
MoCA	27.0 ± 2.5
FOG (Yes/No)	8/52
Falls in last year (none/once/twice/three or more)	39/10/7/4
H&Y (I/II/III)	I: 4, II: 46, III: 10

### Clinical Validation

Good to excellent agreement was observed for mean gait characteristics during baseline walking. ICC_(2,1)_ from 0.849 (stride length) to 0.968 (cadence), and PCC 0.863 (gait speed) to 0.973 (cadence), Table 2. Temporal characteristics, step time, stride length and gait speed showed strongest agreement with minimal error (MAEs ≤ 0.044). Spatial characteristics (gait speed, stride length) demonstrated slightly lower but good agreement (ICC_(2,1)_ ≥ 0.849). During cued walks, agreement remained good to excellent. ICC_(2,1)_ ranged from 0.868 (stride length) to 0.986 (cadence), with PCCs 0.863–0.984. Temporal characteristics again performed best, while spatial showed lower agreement but maintained good reliability (ICC_(2,1)_ ≥ 0.868; MAEs ≤ 0.043). Bland-Altman analysis confirmed close agreement, with negligible bias for all characteristics; participant-level limits of agreement were ± 0.11 m/s (gait speed), ± 10.6 cm (stride length), and ± ~ 2% (CV_stride−time_) (supplementary Table S1, Table S2 and Figure S2).

**Table 2:** Clinical validation for CuePD gait characteristics compared to a reference standard

		Mean			Variability			Asymmetry		
Cue	Characteristic	ICC_(2,1)_ [95% CI]	PCC	MAE	ICC_(2,1)_ [95% CI]	PCC	MAE	ICC_(2,1)_ [95% CI]	PCC	MAE
BL	Step time (s)	0.929 [0.856, 0.966]	0.936	0.008	0.786 [0.594, 0.894]	0.859	0.012	0.821 [0.654, 0.912]	0.829	0.009
	Cadence (steps/min)	0.968 [0.934, 0.985]	0.973	0.845	—	—	—	—	—	—
	Stride length (m)	0.849 [0.705, 0.925]	0.865	0.026	0.229 [− 0.143, 0.545]	0.320	0.019	0.196 [− 0.177, 0.520]	0.311	0.012
	Gait speed (m/s)	0.861 [0.726, 0.931]	0.863	0.044	0.241 [− 0.130, 0.553]	0.338	0.021	0.246 [− 0.125, 0.557]	0.383	0.011
ME	Step time (s)	0.946 [0.889, 0.974]	0.951	0.007	0.777 [0.580, 0.890]	0.856	0.013	0.718 [0.483, 0.857]	0.740	0.012
	Cadence (steps/min)	0.980 [0.958, 0.991]	0.984	0.844	—	—	—	—	—	—
	Stride length (m)	0.868 [0.739, 0.935]	0.865	0.026	0.346 [− 0.007, 0.622]	0.470	0.014	0.224 [− 0.148, 0.541]	0.304	0.010
	Gait speed (m/s)	0.888 [0.777, 0.945]	0.881	0.041	0.309 [− 0.052, 0.596]	0.430	0.018	0.320 [− 0.046, 0.610]	0.490	0.010
IM	Step time (s)	0.938 [0.873, 0.970]	0.943	0.007	0.797 [0.614, 0.900]	0.872	0.011	0.702 [0.457, 0.848]	0.724	0.010
	Cadence (steps/min)	0.986 [0.970, 0.993]	0.982	0.808	—	—	—	—	—	—
	Stride length (m)	0.868 [0.739, 0.935]	0.863	0.025	0.357 [0.006, 0.630]	0.461	0.015	0.218 [− 0.154, 0.536]	0.293	0.012
	Gait speed (m/s)	0.875 [0.753, 0.939]	0.865	0.043	0.296 [− 0.067, 0.586]	0.428	0.020	0.305 [− 0.062, 0.599]	0.473	0.011
VM	Step time (s)	0.932 [0.862, 0.967]	0.939	0.007	0.807 [0.632, 0.904]	0.875	0.011	0.720 [0.486, 0.858]	0.739	0.009
	Cadence (steps/min)	0.985 [0.968, 0.993]	0.981	0.821	—	—	—	—	—	—
	Stride length (m)	0.904 [0.807, 0.953]	0.877	0.022	0.347 [− 0.006, 0.623]	0.449	0.015	0.229 [− 0.143, 0.544]	0.302	0.011
	Gait speed (m/s)	0.915 [0.828, 0.959]	0.886	0.037	0.307 [− 0.054, 0.594]	0.439	0.017	0.319 [− 0.047, 0.609]	0.487	0.009

No values for cadence variability and asymmetry as cadence is represented by total number of steps over a period e.g., 1-min. MAE = mean absolute error (CuePD vs. reference) in each characteristic’s units; errors are absolute, computed per stride and averaged within participant

Variability showed inconsistent agreement. At baseline, step time variability demonstrated good agreement (ICC_(2,1)_ = 0.786). Spatial variability was poor (ICC_(2,1)_ ≤ 0.241). Under cued conditions, step time variability remained good (ICC_(2,1)_ ≥ 0.777), Table 2. For asymmetry, step time showed the strongest agreement at baseline (ICC_(2,1)_ = 0.821), whereas other temporal and spatial characteristics were poor. During cued walking, step time asymmetry declined to moderate agreement (ICC_(2,1)_ 0.702–0.720), and all other asymmetry characteristics remained poor, indicating limited agreement. Consequently, its single-sensor form should not yet be used for spatial variability or asymmetry metrics clinically. See supplementary Table S1 for additionally gait characteristics (e.g., swing time).

### Acute Gait Responses to Cues

From songs within the selected music genres, 29 chose pop, 15 chose rock, 6 chose disco, 4 chose country, 5 chose funk, and 1 chose R&B music. Descriptive statistics for each gait characteristic by cue type, including median and mean changes (both relative and absolute), standard deviation (SD), and the minimum and maximum observed changes are in Table 3. 75% of PwP had improved across all gait characteristics with personalised VM.

**Table 3:** Descriptive statistics and responsiveness by cue type

Characteristics	Cue	Median (%/log)	Median (Δ)	SD (%/log)	SD (Δ)	Min (%/log)	Min (Δ)	Max (%/log)	Max (Δ)	% of PwP improved	Mean (%/log)	Mean (Δ)	SRM
Cadence (steps/min)	ME	6.030	6.632****	4.898	4.776	−6.734	−6.888	27.725	23.437	87	5.562	5.832	1.135
	IM	5.805	6.175****	5.233	5.437	−18.239	−20.948	24.284	20.528	92	4.976	5.191	0.951
	VM	5.976	6.393****	4.144	4.050	−2.843	−3.109	25.214	21.314	92	5.595	5.878	1.350
Gait Speed (m/s)	ME	7.280	0.073****	11.207	0.097	−15.601	−0.161	53.847	0.352	88	8.779	0.080	0.783
	IM	10.753	0.109****	11.490	0.106	−28.812	−0.314	36.120	0.319	78	9.739	0.088	0.848
	VM	12.711	0.123****	10.046	0.088	−10.098	−0.087	41.822	0.343	92	12.702	0.115	1.264
Stride length (cm)	ME	2.815	3.000**	8.392	8.600	−21.716	−21.800	20.445	23.400	70	3.117	3.300	0.371
	IM	6.062	6.200****	8.093	8.300	−17.356	−17.800	24.916	23.300	73	4.506	4.700	0.557
	VM	7.915	8.000****	7.740	8.000	−15.592	−14.200	31.684	29.600	80	6.758	7.000	0.873
CV_stride−time_ (%)	ME	0.012	0.029	0.458	1.396	−0.762	−1.533	1.326	5.613	48	0.047	0.259	0.186
	IM	0.039	0.089	0.472	1.581	−0.978	−2.895	1.396	6.169	48	0.048	0.279	0.177
	VM	−0.152	−0.413***	0.362	0.827	−0.890	−2.419	0.776	1.937	75	−0.181	−0.418	−0.505

Asterisks denote significance of within-cue change from baseline (paired t-test or Wilcoxon signed-rank test, depending on normality): * *p* ≤ 0.05, ** *p* ≤ 0.01, *** *p* ≤ 0.001, **** *p* ≤ 0.0001. Relative change is expressed as percentage change from baseline, except for stride time CV, which is expressed as the natural log of the ratio (log cue/baseline). Absolute change (Δ) is reported in the original units: m/s for gait speed, cm for stride length, steps/min for cadence, and % for CV

#### Cadence

**Fig. 1:**
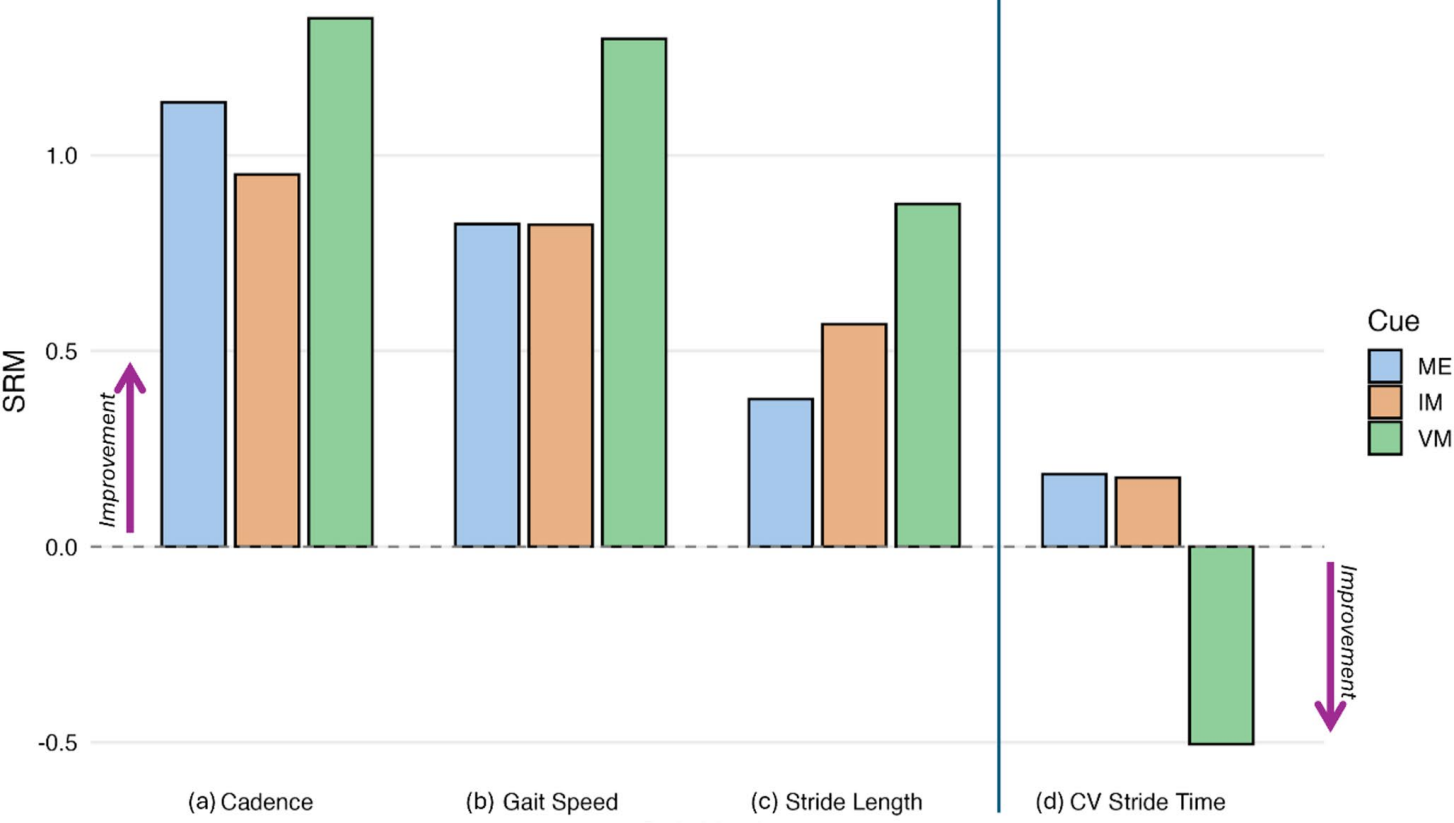
SRM (standardised response mean) across gait characteristics by cue type. Positive for a, b and c = improvement for cadence, gait speed and stride length, respectively. Negative for d = improvement for CV_stride time_ (i.e., gait variability reduced).Vocal music (VM) was the most effective to improve all gait characteristics, compared to instrumental music (IM), and metronome (ME)

Mean percentage changes were 5.562% (ME), 4.976% (IM), and 5.595% (VM), with SRMs of 1.135, 0.951, and 1.350, respectively (Table 3; Fig. 1). Mauchly’s test indicated violation of sphericity (W = 0.849, *p* = 0.010), and Greenhouse–Geisser-corrected results were therefore interpreted. The mixed ANOVA and ART showed no significant main effect of cue type, walk order, or cue×walk order interaction (Table 4).

**Table 4:** Cueing and walk order effects on gait characteristics

			Mixed ANOVA (Cue, Walk Order, Cue x Walk Order Interaction)				ART (Cue, Walk Order, Cue x Walk Order Interaction)			Significant post hoc comparisons
Characteristics	Main effect		F	DoF	*p*	ges	F	df	*p*	Paired T-Test/Wilcoxon (Bonferroni)
*Cadence*	Cue		1.64	1.74, 99.01	0.202	0.004	0.91	2, 114	0.407	-
	Walk Order		1.26	2, 57	0.290	0.037	1.35	2, 57	0.268	-
	Interaction		0.74	3.47, 99.01	0.548	0.003	1.21	4, 114	0.312	**-**
*Gait Speed*	Cue		5.42	1.61, 91.95	**0.006**	0.024	6.74	2, 114	**0.002**	VM > ME (p = **0.027**); VM > IM (p = **0.005**)
	Walk Order		0.97	2, 57	0.387	0.024	0.59	2, 57	**0.558**	-
	Interaction		1.93	3.23, 91.95	0.126	0.017	1.15	4, 114	0.335	-
*Stride Length*	Cue		6.21	1.57, 89.56	**0.003**	0.035	6.42	2, 114	**0.002**	VM > ME (*p* = 0.**009**); VM > IM (*p* = 0.**011**)
	Walk Order		0.33	2, 57	0.717	0.008	0.32	2, 57	**0.729**	-
	Interaction		2.50	3.14, 89.56	0.062	0.028	1.64	4, 114	0.169	-
*CV Stride Time*	Cue		14.59	2, 114	**< 0.001**	0.062	13.74	2, 114	**< 0.001**	VM < ME (p **< 0.001**); VM < IM (p **< 0.001**)
	Walk Order		1.54	2, 57	0.223	0.039	1.77	2, 57	0.179	-
	Interaction		2.07	4, 114	0.090	0.018	1.94	4, 114	0.109	-

Assumptions were formally assessed. Two-way mixed ANOVA was used for all characteristics, with Greenhouse–Geisser correction applied where required; F, degrees of freedom (DoF), p, and ges reported for all main effects and interactions. ART was used as a non-parametric sensitivity/robustness check. Similarly, Bonferroni-adjusted post hoc comparisons are reported only where a significant main effect of cue is observed. *ME* metronome, *IM* instrumental music, *VM* vocal music, *ges* generalized eta squared (effect size for ANOVA effects). Significant results are shown in bold

#### Gait Speed

Mean absolute changes were 0.080 m/s (ME), 0.088 m/s (IM), and 0.115 m/s (VM), with SRMs of 0.783, 0.848, and 1.264, respectively (Table 3; Fig. 1). Mauchly’s test indicated violation of sphericity (W = 0.760, *p* < 0.001), and Greenhouse–Geisser-corrected results were therefore interpreted. The mixed ANOVA (p[GG] = 0.006) and ART (*p* = 0.002) showed a significant main effect of cue type, with no significant main effect of walk order or cue×walk order interaction. As the paired cue differences were non-normally distributed, Bonferroni-adjusted Wilcoxon signed-rank post hoc comparisons were performed, showing significantly greater values during VM than ME (*p* = 0.027) and IM (*p* = 0.005), whereas ME and IM did not differ significantly (Table 4).

#### Stride Length

Mean absolute changes were 3.3 cm (ME), 4.7 cm (IM), and 7.0 cm (VM), with SRMs of 0.371, 0.557, and 0.873, respectively (Table 3; Fig. 1). Mauchly’s test indicated violation of sphericity (W = 0.727, *p* < 0.001), and Greenhouse–Geisser-corrected results were therefore interpreted. The mixed ANOVA (p[GG] = 0.003) and ART (*p* = 0.002) showed a significant main effect of cue type, with no significant main effect of walk order or cue×walk order interaction. As the paired cue differences were non-normally distributed, Bonferroni-adjusted Wilcoxon signed-rank post hoc comparisons were performed, showing it was significantly greater during VM than during ME (*p* = 0.009) and IM (*p* = 0.011), whereas ME and IM did not differ significantly (Table 4).

#### Cv_stride−time_

Mean absolute changes were + 0.259% (ME), + 0.279% (IM), and − 0.418% (VM), with SRMs of 0.186, 0.177, and − 0.505, respectively (Table 3; Fig. 1). Sphericity was not violated, and the mixed ANOVA showed a significant main effect of cue type (*p* < 0.001), with no significant main effect of walk order or cue × walk order interaction. As the paired cue differences were normally distributed, Bonferroni-adjusted paired t-test post hoc comparisons were performed, showing it was significantly lower during VM than during ME (*p* < 0.001) and IM (*p* < 0.001), whereas ME and IM did not differ significantly (Table 4). See supplementary Figure S3 for box-plot representation of changes between different cues.

#### First Cue Carryover Effect

Carryover effects of the initial cue exposure were evaluated by comparing retention scores at the first washout walk (WO3) across participants grouped by first cue type. No statistically significant differences in retention were found between first-cue groups for cadence (H = 0.26, *p* = 0.877), gait speed (F = 0.91, *p* = 0.407), stride length (H = 2.68, *p* = 0.262), or CV_stride−time_ (H = 3.07, *p* = 0.216). Findings suggest that performance at the first washout walk was not differentially influenced by the type of cue presented first, indicating no clear short-term carryover effect of the initial cue condition (supplementary Table S3).

### Acceptance, Usability and Musicality

Of 60 PwP, 54 (90%) identified VM as their preferred cue, 4 preferred Me, 2 reported no preference, and none-preferred IM. VM was described as more enjoyable, natural, and motivating, while the metronome was often perceived as boring or clinical. Participants reported VM improved pacing, rhythm, and ease of movement. Those preferring ME found it easier to follow but still considered VM more engaging. The SUS score (85.92) indicates excellent exceeding the 83.28 average for physical activity apps [28], with 85% reporting they would use CuePD frequently (supplementary Table S4).

Musicality versus cue adherence was assessed with the Gold-MSI via Spearman correlations. No significant associations were found between Gold-MSI scores and adherence error for VM or IM cueing after Bonferroni correction. Sensitivity analysis using signed error also showed no significant relationships (supplementary Table S5). Additionally, no significant correlations were observed between Gold-MSI scores and cue-related changes in gait characteristics for either VM or IM after correction (supplementary Table S6 and Table S7).

## Discussion

This study is the first to investigate the clinical validity and effectiveness of a single device approach to retrain gait and acutely improve characteristics related to fall risk in PwP. CuePD robustly captures clinical gait characteristics that could be useful within fall risk models and effectively provides a meaningful and personalised intervention to improve gait characteristics synonymous with fall risk. CuePD reduced CV_stride−time_ while increasing stride length and gait speed which target gait deficits relevant to PwP, that could deliver an adequate benefit-to-burden ratio in PwP.

Many digital gait interventions have been proposed with varying success [33]. Wearable and smartphone-based systems have shown that real-time feedback can support improvements in gait. Specifically, biofeedback interventions, have shown benefits in selected characteristics [34–36], while a multifaceted smartphone-based systems has improved gait performance and engagement in home environments [37]. Prominent approaches typically use auditory cueing, whereby PwP are tasked to step to a metronome, and although that approach can be beneficial [38], it has limited long-term engagement and adaptability to the person’s daily or weekly needs [39, 40]. Realistically, wearables implementing metronome-based cues and/or with more sophisticated (i.e., costly) peripheral technologies have little pragmatic use and fail to holistically conform to MDS Task Force recommendations i.e., a homogenous platform. Here, CuePD conforms to an unmet need as identified by an expert task force for technology in PwP gait retraining to fulfil verification, analytical validation and clinical validation [19].

### Clinical Validity

There was good to excellent agreement (ICC_(2,1)_ ≥ 0.849) for mean temporal and spatial characteristics, like other work [41–43]. We interpreted our LoA against published minimal clinically important differences (MCIDs) derived from an instrumented walkway in PwP [44]. The LoA describe how closely CuePD and the reference wearable agree, comparing them with exemplar MCID values (albeit from a walkway) provides some insight as to whether the app can detect a clinical meaningful change. Encouragingly, bias was negligible for all characteristics, showing no systematic over- or under-estimation. Participant-level gait-speed limits (± 0.11 m/s) closely approached the velocity MCID (0.082 m/s), meaning the app resolves gait speed to near the smallest clinically meaningful change, and stride-length limits (± 10.6 cm) fell within the stride-length MCID range (1.8–13.5 cm), supporting CuePD for measuring mean gait at the participant level. Only CV_stride−time_ limits (± ~ 2%) exceeded the comparison MCID range (0.07–0.92%), which could be expected for a variability measure derived from a device on the lower-back (compared to wearables at the feet). Importantly, limits halved when aggregated by participant (rather than per 1-minute walk). As CuePD is designed for continuous rather than single-walk monitoring, real-world precision is expected to improve further, plausibly within MCID thresholds across all characteristics.

Variability (presented here as CV) is sensitive to methodological differences than mean gait measures. As a second-order statistic, CV_stride−time_ is strongly influenced by the precision of gait event detection, the number of strides included, and the processing pipeline, resulting in greater measurement uncertainty. Specifically, the comparison MCIDs were derived using an instrumented walkway, whereas our estimates were obtained from a lower-back wearable, and variability measures are known to be less transferable across measurement systems than mean spatial and temporal characteristics. Equally, the poor-average agreement observed for variability and asymmetry characteristics is not unexpected and comparable to previous findings from wearables at different anatomical locations i.e., lower back and feet [41, 42]. Those characteristics are also more sensitive to small errors in gait-event detection, stride segmentation, sensor placement, and short trial duration than mean gait characteristics. Variability and asymmetry are second-order characteristics, so small differences between the smartphone and reference system can be amplified. Therefore, while CuePD appears suitable for quantifying mean gait characteristics, the current results suggest that spatial variability and asymmetry characteristics should be interpreted cautiously and should not yet be considered clinically interchangeable with the reference system. Although placement of a device on the feet is more optimal for absolute gait sequence timing [7, 45], it is unrealistic/unlikely that attachment location would be viable for continuous use.

If a smartphone is to be used as the optimal single device then a more convenient carry location is required e.g., pocket [8]. Currently, CuePD implements a validated gait algorithm that is optimised for use at the lower back [27, 41] and recommended for use in PwP [46]. Alternative deep learning artificial intelligence based algorithms exist including one that quantifies gait from placement of a smartphone in the pocket [47]. The ability to quantify gait from a more convenient wear location could aid adherence and the reference study shows promise for asymmetrical gait assessment in other neurological conditions e.g., stroke survivors, but such an approach needs clinical validation in PwP. The V3 framework supports robust evidence generation for implementation, while other frameworks emphasise usability and stakeholder involvement [48, 49]. As CuePD is intended as a personalised intervention for PwP, we considered this group the primary stakeholder and assessed feedback via the SUS [28], with positive outcomes.

### Personalised Cues

VM elicited the closest cadence increase to the + 10% target (5.595%, Δ = 5.878 steps/min), but the mixed ANOVA showed no significant effect of cue type on cadence (*p* = 0.202) and the degree of cadence adherence was unrelated to, and not a driver of gait gains. Despite falling short, cadence increase under VM was sufficient to support significant gains, compared to ME. Significant improvement across gait characteristics is notable when compared to other ME-based studies, which often fail to demonstrate significant gains in gait speed, stride length, and gait variability [23, 50, 51]. Interestingly, Sowalsky et al. [52] used a personalised fractal metronome approach to show improved stride time fluctuations compared to regular metronome or music. Regardless, the long term adherence to a metronome based tone is likely to be limited [14].

ME and IM produced inconsistent effects on CV_stride−time_, where, on average, PwP exhibited increased variability, Fig. 1. The significantly greater reduction in CV_stride−time_ with VM than IM suggests that vocals/lyrics and perhaps greater familiarity via groove [52], may be central to music-based cueing. Music may enhance recognition and emotional engagement, with neuroimaging implicating memory, emotion and motor regions via the dopaminergic pathway, and peak musical pleasure linked to higher striatal dopamine release [53–55]. However, response could be further amplified when music aligns with personal preference and familiarity, as peak interest has been associated with higher dopamine release in the striatum, a critical component of the neural motor and reward system.

Preference and familiarity are distinct constructs, where preferred genre (broad style), preferred song (self-selected track), familiarity (prior exposure), and enjoyment (affective response) may each contribute differently to cueing response [56–58]. Familiar, preferred music also can reduce cognitive demand while heightening pleasure, supporting interventions that encourage engagement and allow autonomous, personally meaningful music choice [59, 60]. Although familiarity does not enhance walking speed in healthy adults, any dopaminergic response may be amplified in PwP given their characteristic dopaminergic dysfunction [61]. This could explain why a higher percentage of participants improved with VM (92% cadence, 92% gait speed, 80% stride length, 75% CV_stride−time_), compared to prior studies using non-personalised (preference) music, where participants were either assigned generic music or only allowed to select a genre rather than a preferred song [51, 62–65]. It was previously hypothesised [62] that the lack of response was dependent on rhythmic ability or musicality, evidenced by 56.4% of participants showing no improvement in gait speed. Yet, results show markedly higher response rates regardless of musicality, suggesting factors beyond rhythmic ability (i.e., music preference/enjoyment, familiarity, emotional connections), may play a significant role in determining an individual’s positive responsiveness to cueing.

Accordingly, observed improvements in fall risk related outcomes are likely driven by the combination of moderately elevated tempo, lyrical familiarity, personal music preference, and emotional salience, highlighting a multifactorial mechanism through which VM enhances motor performance. Against the same PwP MCIDs, the VM-induced changes were clinically meaningful: gait speed rose by 0.115 m/s (exceeding 0.082 m/s), stride length by 7.0 cm (within 1.8–13.5 cm), and CVstride-time fell by 0.418% (within 0.07–0.92%). Findings support the efficacy of personalised VM cueing, particularly when delivered at + 10% of baseline tempo. By aligning biomechanically relevant pacing with emotionally engaging and familiar music, CuePD simultaneously targets motor and enjoyment pathways. Consequently, VM via preferred song choice presents a compelling intervention for improving gait in PwP.

### Usability

#### Auditory Cueing Preferences and Usability

The overwhelming preference for VM (90%) over ME (7%) underscores the importance of enjoyment in gait interventions. Participants consistently described music as more engaging, natural, and motivating, while ME was often perceived as monotonous or clinical. These impressions were not only qualitative but seemed to influence walking behaviour, where many individuals reported walking more confidently and rhythmically with music, suggesting a potential motivational and attentional advantage. Interestingly, while a small minority (7%) found ME easier to follow, even these individuals acknowledged the superior enjoyability of music. This distinction highlights an important consideration, ease of synchronisation may not equate to user preference, and the affective dimension of auditory cues may play a critical role in long-term adherence to walking programs. The high SUS score (85) reinforces the practicality of CuePD and its potential for continued use outside of a laboratory context.

#### Musical Sophistication and Cueing Response

Findings did not support participant musicality would enhance the ability to synchronise with rhythmic cues. Across all Gold-MSI domains, there were no significant associations between musical sophistication and cue adherence or changes in gait characteristics. This suggests that cueing efficacy may not depend on musical training or perceptual skills, making the intervention more broadly accessible and effective.

A previous study [62] suggested that higher musical sophistication may buffer against dual-task interference by facilitating more automatic rhythmic entrainment. The discrepancy between findings may reflect differences in sample characteristics, cueing implementation, or the cognitive demands placed on participants. Notably, many participants described music as requiring less conscious effort compared to ME, implying that perceived cognitive load, rather than musicality, might modulate cueing response. Together, these findings highlight that the affective and cognitive properties of auditory cues may be as critical as their rhythmic properties. Furthermore, these analyses were sensitive to moderate-to-large associations but could not exclude smaller ones, consistent with [62]. While musical sophistication may not directly influence outcomes, the choice of cueing modality clearly shapes user experience, motivation, and possibly engagement with the rhythm itself. Future work should further explore whether tailoring cueing to individual preferences, rather than musical background, yields better adherence and functional outcomes.

#### Device Trade-Off

Comparable examples of personalised gait intervention via smartphone-based cueing exist with evidence to suggest their positive use to improve gait in PwP. Zoetewei et al. [66] implemented ME cueing (with a vocal command) to overcome real time detection of freezing of gait. Cochen de Cock et al. [18] elicited an increase of spontaneous cadence via PwP selecting from 2 of 6 genres across 285 songs whereby song tempo was altered by a time-stretching algorithm influenced by real-time gait assessment. In contrast, Porciuncula et al. [67] used a bespoke rhythmic auditory stimulation closed loop system/device (MR-005, MedRhythms, Inc, Portland, ME, USA). However, a major limitation of all those studies is use of peripheral wearables to be worn on both ankles or both feet, limiting their wider deployment due to increased cost (i.e., multi-device) and likelihood of technical hurdles that may be needed to be overcome with e.g., connectivity, charging. CuePD is a single device approach on a person’s own smartphone to greatly reduce cost and target scalability.

### Limitations and Future Work

Data were collected in a lab, which does not represent real-world walking or ecological validity of CuePD. Controlled conditions also may not fully capture the variability, distractions, or terrain changes [68] that individuals encounter in daily life [69]. Participants were mostly tested ON (58/60), Hoehn & Yahr II, and non-fallers, with freezing rare and cognitive impairment excluded (MoCA < 21); findings may not generalise to OFF states, advanced PD, recurrent fallers, freezing, or cognitive impairment. Accuracy of gait estimation may also depend on consistent device orientation and placement, as variations in how the device is worn can affect sensor alignment and introduce errors [70]. This could be reduced by providing clear instructions on how to wear the device consistently, although some variation in real-world use is likely to remain. Furthermore, use of an oval circuit, means turns may have influenced agreement between CuePD and the reference wearable [42]. The belt-mounted L5 setup also differs from real-world use, though cueing could run from a pocket.

Washout may not have fully removed residual carryover, fatigue or learning effects. As a single-session lab study, benefits may partly reflect novelty, and long-term adherence, retention and effects on falls remain untested. As such, the observed effects of auditory cueing may differ in free-living contexts. This underscores the need to evaluate CuePD’s effectiveness during community-based and/or home-based use over extended periods. Accordingly, our future work (from July 2026) will be a 12-week pilot randomised controlled trial to examine ecological validity of CuePD in PwP, evaluating gait characteristics alongside duration of use and engagement over extended real-world use.

### Conclusion

CuePD is a novel gait retraining app that conforms to the MDS Task Force on Technology recommendations: a single device that balances patient burden with clinical utility and a personalised approach to target deficits relevant to PwP. The CuePD approach may offer an appropriate and effective resource to address impaired gait, a known contributor to falls, having broad appeal due to its use on already distributed technology. CuePD is clinically valid to reliably measure mean gait characteristics, while showing acute effectiveness for retraining gait. Moreover, CuePD provides cueing via contemporary and preferred music choices which suggests a more engaging approach to gait retraining in PwP, at least in the short term. High usability ratings, and strong participant preference for music cues regardless of musical background suggest CuePD as a plausible and likeable tool in PwP. Beyond a standalone intervention, CuePD’s smartphone architecture could possibly integrate within digital care pathways, enabling remote monitoring and linkage to clinical workflows for decentralised PD management. Therefore, CuePD may offer tangible and pragmatic real-world potential to improve gait; however, its ecological validity and long-term clinical effectiveness, including whether it reduces falls, remain to be tested in future trials beyond the lab.

## Supplementary Information

Below is the link to the electronic supplementary material.

Supplementary Material 1 (DOCX 591 KB)

## Data Availability

The data are available upon reasonable request from the corresponding author.
