# Supplementary material for: A Digital Cueing Intervention for Parkinsonian Gait: Laboratory-Based Clinical Validation and Acute Gait Responses
Source: J Med Syst. 2026 Jul 11;50(1):112. doi: 10.1007/s10916-026-02441-x (PMC13356047; doi:10.1007/s10916-026-02441-x)
Supplement: Supplementary file 1 — Supplementary Material 1 (DOCX 591 KB) [file 10916_2026_2441_MOESM1_ESM.docx]

**Supplementary online material**


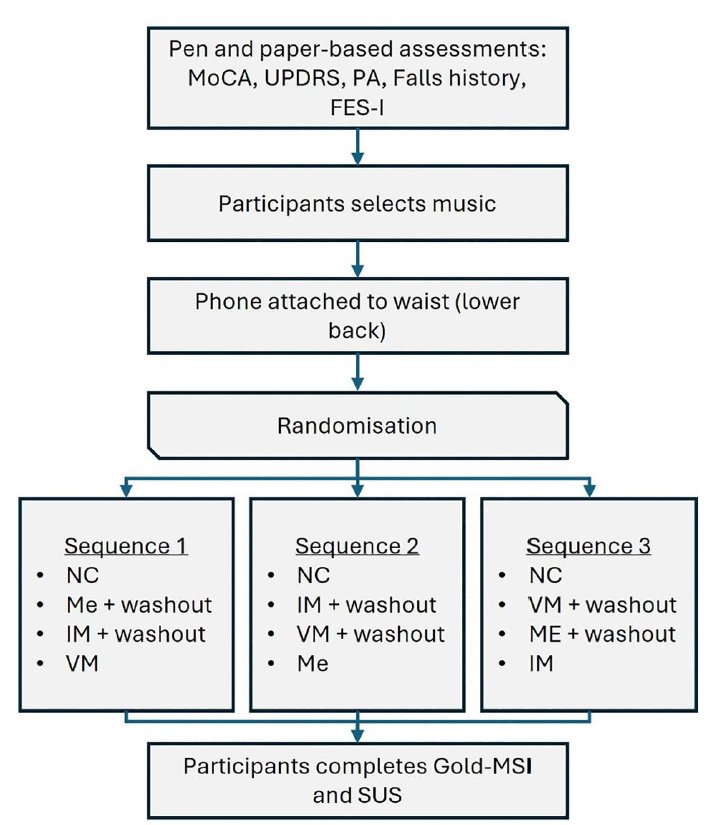


Figure S1: Participant flow diagram

**Table S1. Additional gait characteristics validation**

|  |  | Mean | | | Variability | | | Asymmetry | | |
| --- | --- | --- | --- | --- | --- | --- | --- | --- | --- | --- |
| Cue | Gait characteristic | ICC_2,1_ | PCC | MAE | ICC_2,1_ | PCC | MAE | ICC_2,1_ | PCC | MAE |
| Baseline | Stride time (s) | 0.906 | 0.918 | 0.009 | 0.436 | 0.671 | 0.015 | 0.328 | 0.514 | 0.014 |
|  | Stance time (s) | 0.887 | 0.897 | 0.009 | 0.281 | 0.424 | 0.016 | 0.346 | 0.536 | 0.013 |
|  | Swing time (s) | 0.866 | 0.878 | 0.009 | 0.514 | 0.696 | 0.012 | 0.286 | 0.492 | 0.012 |
| Me | Stride time (s) | 0.954 | 0.960 | 0.006 | 0.504 | 0.719 | 0.012 | 0.386 | 0.455 | 0.012 |
|  | Stance time (s) | 0.924 | 0.944 | 0.007 | 0.235 | 0.399 | 0.016 | 0.281 | 0.439 | 0.011 |
|  | Swing time (s) | 0.894 | 0.902 | 0.007 | 0.286 | 0.445 | 0.013 | 0.407 | 0.605 | 0.011 |
| IM | Stride time (s) | 0.950 | 0.957 | 0.007 | 0.485 | 0.719 | 0.014 | 0.407 | 0.477 | 0.011 |
|  | Stance time (s) | 0.935 | 0.957 | 0.006 | 0.214 | 0.397 | 0.018 | 0.399 | 0.615 | 0.010 |
|  | Swing time (s) | 0.892 | 0.901 | 0.007 | 0.266 | 0.425 | 0.014 | 0.275 | 0.446 | 0.008 |
| VM | Stride time (s) | 0.971 | 0.987 | 0.050 | 0.503 | 0.735 | 0.011 | 0.385 | 0.467 | 0.011 |
|  | Stance time (s) | 0.922 | 0.942 | 0.007 | 0.217 | 0.389 | 0.017 | 0.268 | 0.443 | 0.009 |
|  | Swing time (s) | 0.891 | 0.910 | 0.007 | 0.255 | 0.420 | 0.014 | 0.398 | 0.619 | 0.012 |

**Table S2. Bland-Altman agreement between CuePD and the reference standard for each gait outcome, by walk condition.**

| **Cue** | **Gait Characteristic** | **Bias** | **SD of difference** | **Lower 95% LoA** | **Upper 95% LoA** |
| --- | --- | --- | --- | --- | --- |
| Baseline | Cadence (steps/min) | +0.20 | 2.54 | -4.79 | +5.19 |
|  | Gait speed (m/s) | -0.015 | 0.108 | -0.227 | +0.197 |
|  | Stride length (cm) | -1.00 | 10.78 | -22.14 | +20.14 |
|  | CV_stridetime_ (%) | +0.10 | 2.07 | -3.96 | +4.16 |
| ME | Cadence (steps/min) | +0.20 | 2.09 | -3.90 | +4.30 |
|  | Gait speed (m/s) | -0.015 | 0.105 | -0.220 | +0.190 |
|  | Stride length (cm) | -1.00 | 10.18 | -20.96 | +18.96 |
|  | CV_stridetime_ (%) | +0.10 | 2.13 | -4.07 | +4.27 |
| IM | Cadence (steps/min) | +0.15 | 1.82 | -3.41 | +3.71 |
|  | Gait speed (m/s) | -0.012 | 0.108 | -0.224 | +0.200 |
|  | Stride length (cm) | -0.80 | 10.17 | -20.73 | +19.13 |
|  | CV_stridetime_ (%) | +0.08 | 2.27 | -4.37 | +4.53 |
| VM | Cadence (steps/min) | +0.10 | 1.83 | -3.48 | +3.68 |
|  | Gait speed (m/s) | -0.010 | 0.082 | -0.171 | +0.151 |
|  | Stride length (cm) | -0.60 | 8.11 | -16.50 | +15.30 |
|  | CV_stridetime_ (%) | +0.05 | 1.85 | -3.58 | +3.68 |


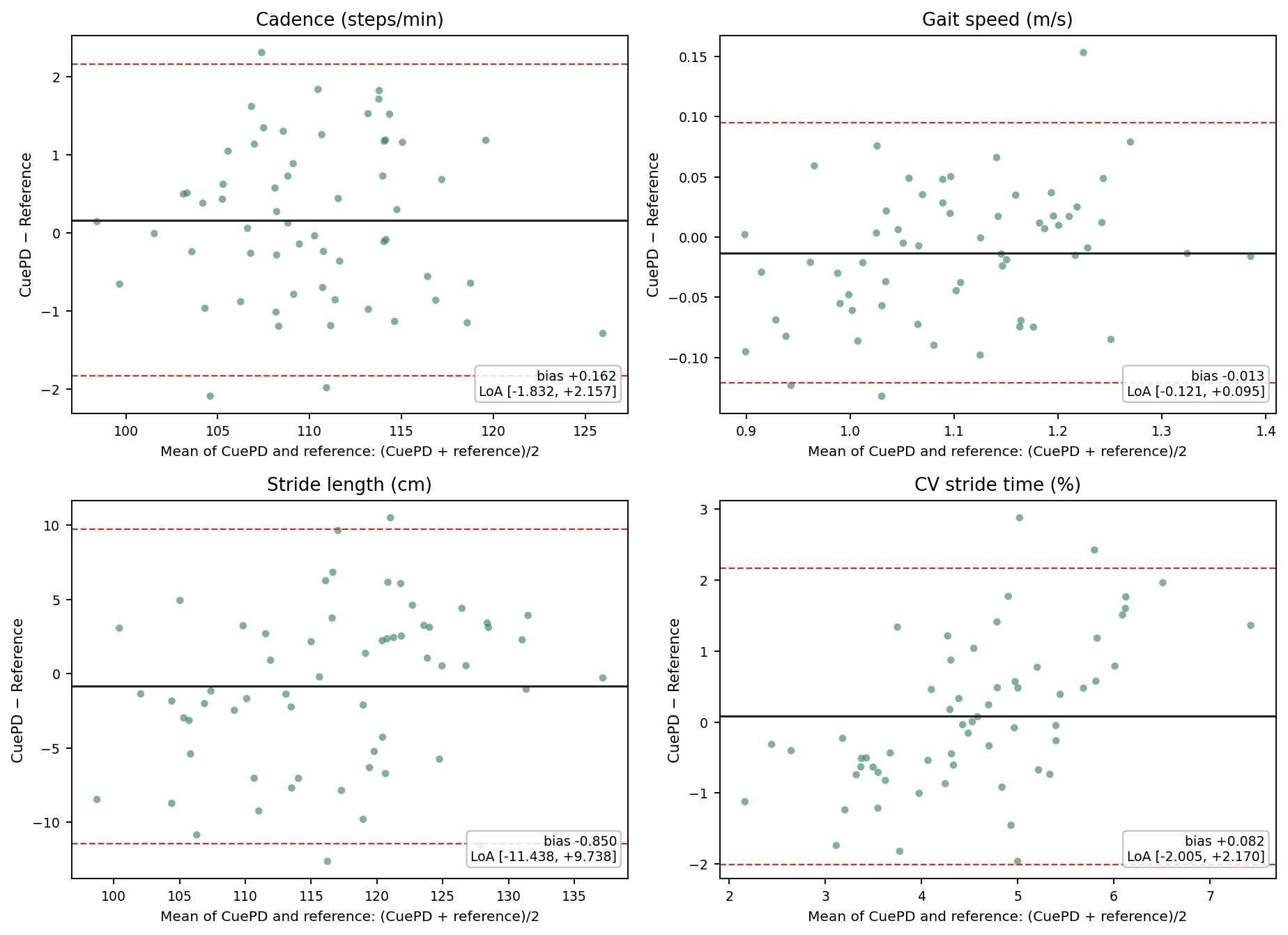


**Figure S2. Participant-level Bland-Altman agreement between CuePD and the reference standard for each gait outcome (n = 60). Each point is one participant (values averaged across all walks); the solid line shows mean bias and dashed lines the 95% limits of agreement.**


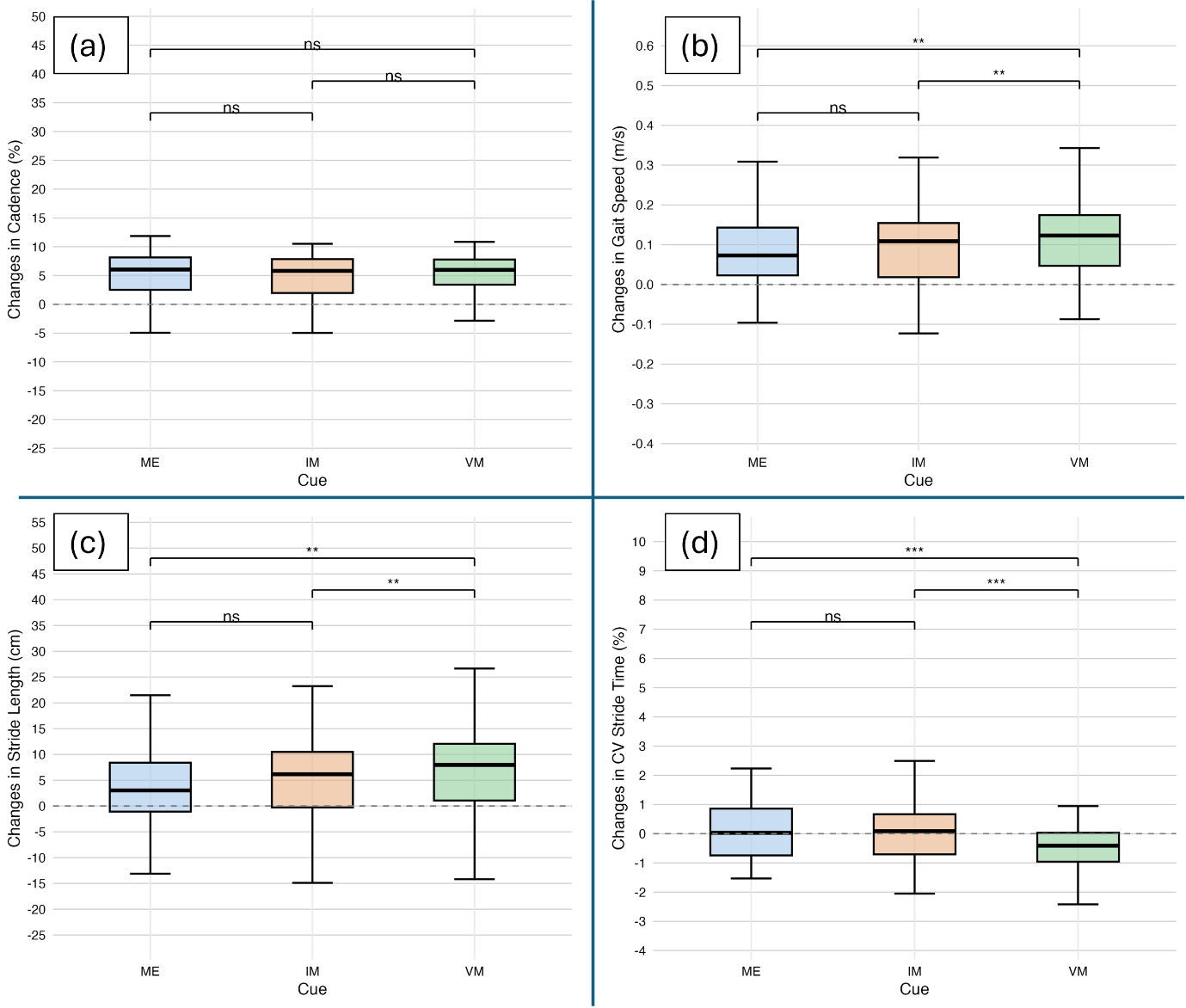


**Figure S3. Changes in (a) cadence, (b) gait speed, (c) stride length and (d) CV_stride-time_**

| ***Outcome*** | ***Test*** | ***Statistic*** | ***p*** | ***First cue*** | ***Mean*** | ***SD*** | ***Min*** | ***Median*** | ***Max*** |
| --- | --- | --- | --- | --- | --- | --- | --- | --- | --- |
| Cadence (SPM) | Kruskal-Wallis | H = 0.26 | 0.88 | Me | -2.94 | 3.00 | -8.29 | -1.95 | 1.04 |
|  |  |  |  | IM | -3.64 | 3.74 | -11.9 | -3.00 | 2.53 |
|  |  |  |  | VM | -3.04 | 3.29 | -6.89 | -4.18 | 3.59 |
| Gait speed (m/s) | One-way ANOVA | F = 0.91 | 0.41 | Me | -2.98 | 7.14 | -17.2 | -1.76 | 12.4 |
|  |  |  |  | IM | -0.94 | 10.2 | -21.4 | 1.66 | 18.9 |
|  |  |  |  | VM | -4.67 | 8.55 | -23.1 | -3.48 | 8.69 |
| Stride length (cm) | Kruskal-Wallis | H = 2.68 | 0.26 | Me | 0.03 | 6.40 | -8.78 | -0.29 | 19.8 |
|  |  |  |  | IM | 2.53 | 8.78 | -11.9 | 3.24 | 23.7 |
|  |  |  |  | VM | -1.46 | 6.73 | -15.6 | -1.59 | 13.7 |
| CV_stride-time_ (log) | Kruskal-Wallis | H = 3.07 | 0.22 | Me | -19.9 | 32.3 | -83.0 | -17.7 | 19.8 |
|  |  |  |  | IM | -30.6 | 40.8 | -164.0 | -28.0 | 21.1 |
|  |  |  |  | VM | -8.61 | 31.5 | -70.6 | -10.4 | 38.6 |

**Table S3. Carryover effects from first cue to subsequent washout walk**

**Table S4. System Usability Scale (SUS) results**

| Q# | Description | Mean raw score | Adjusted  mean  score |
| --- | --- | --- | --- |
| 1 | I think I would like to use this tool frequently. | 4.3 | 3.3 |
| 2 | I found the tool unnecessarily complex. | 1.3 | 3.7 |
| 3 | I thought the tool was easy to use. | 4.5 | 3.5 |
| 4 | I think that I would need the support of a technical person to be able to use this system. | 2.1 | 2.9 |
| 5 | I found the various functions in this tool were well integrated. | 4.2 | 3.2 |
| 6 | I thought there was too much inconsistency in this tool. | 1.4 | 3.6 |
| 7 | I would imagine that most people would learn to use this tool very quickly. | 4.6 | 3.6 |
| 8 | I found the tool very cumbersome to use. | 1.3 | 3.8 |
| 9 | I felt very confident using the tool. | 4.6 | 3.6 |
| 10 | I needed to learn a lot of things before I could get going with this tool. | 3.2 | 1.8 |

| **Sub-scales** | **Metric** | **Correlation (r)** | **95% CI** | **P** |
| --- | --- | --- | --- | --- |
| Active engagement | IM % | 0.07 | -0.18 - 0.33 | 1.00 |
|  | VM % | 0.01 | -0.22 - 0.26 | 1.00 |
| Perceptual abilities | IM % | -0.13 | -0.52 - 0.10 | 1.00 |
|  | VM % | -0.24 | -0.58 - 0.01 | 0.40 |
| Musical training | IM % | -0.21 | -0.51 - 0.05 | 0.70 |
|  | VM % | -0.21 | -0.46 - 0.03 | 0.61 |
| Singing abilities | IM % | 0.02 | -0.24 - 0.23 | 1.00 |
|  | VM % | 0.01 | -0.33 - 0.17 | 1.00 |
| Emotional response | IM % | -0.01 | -0.40 - 0.25 | 1.00 |
|  | VM % | -0.07 | -0.46 - 0.18 | 1.00 |
| General music sophistication | IM % | -0.10 | -0.48 - 0.16 | 1.00 |
|  | VM % | -0.23 | -0.54 - 0.03 | 0.46 |

**Table S5. Gold-MSI subscale scores vs cue adherence error (%) under IM and VM.**

**Table S6. Gold-MSI subscale scores vs change in gait outcomes under IM and VM cueing.**

| Sub-scales | Metric | Cue | Correlation (r) | 95% CI | P |
| --- | --- | --- | --- | --- | --- |
| Active engagement | Gait speed | IM | 0.06 | -0.210 – 0.308 | 0.67 |
|  |  | VM | 0.01 | -0.275 – 0.264 | 0.96 |
|  | Stride length | IM | 0.10 | -0.164 – 0.363 | 0.44 |
|  |  | VM | 0.05 | -0.223 – 0.314 | 0.70 |
|  | CV_stride-time_ | IM | 0.13 | -0.135 – 0.374 | 0.34 |
|  |  | VM | -0.11 | -0.346 – 0.124 | 0.41 |
| Perceptual abilities | Gait speed | IM | 0.04 | -0.195 – 0.274 | 0.76 |
|  |  | VM | 0.01 | -0.251 – 0.251 | 0.96 |
|  | Stride length | IM | 0.00 | -0.259 – 0.237 | 0.98 |
|  |  | VM | -0.08 | -0.376 – 0.180 | 0.53 |
|  | CV_stride-time_ | IM | 0.06 | -0.176 – 0.300 | 0.64 |
|  |  | VM | -0.09 | -0.336 – 0.169 | 0.50 |
| Musical training | Gait speed | IM | 0.15 | -0.115 – 0.392 | 0.27 |
|  |  | VM | 0.14 | -0.115 – 0.373 | 0.29 |
|  | Stride length | IM | 0.10 | -0.166 – 0.354 | 0.44 |
|  |  | VM | 0.09 | -0.175 – 0.341 | 0.48 |
|  | CV_stride-time_ | IM | 0.06 | -0.187 – 0.298 | 0.66 |
|  |  | VM | 0.06 | -0.190 – 0.289 | 0.66 |
| Singing abilities | Gait speed | IM | 0.08 | -0.158 – 0.325 | 0.57 |
|  |  | VM | 0.02 | -0.219 – 0.274 | 0.90 |
|  | Stride length | IM | 0.13 | -0.107 – 0.383 | 0.33 |
|  |  | VM | 0.04 | -0.215 – 0.307 | 0.74 |
|  | CV_stride-time_ | IM | 0.13 | -0.130 – 0.372 | 0.32 |
|  |  | VM | -0.08 | -0.314 – 0.141 | 0.53 |
| Emotional response | Gait speed | IM | 0.00 | -0.247 – 0.242 | 0.98 |
|  |  | VM | 0.04 | -0.207 – 0.280 | 0.77 |
|  | Stride length | IM | 0.05 | -0.202 – 0.284 | 0.70 |
|  |  | VM | 0.04 | -0.201 – 0.263 | 0.78 |
|  | CV_stride-time_ | IM | 0.04 | -0.210 – 0.299 | 0.77 |
|  |  | VM | 0.00 | -0.267 – 0.275 | 0.98 |
| General music sophistication | Gait speed | IM | 0.09 | -0.164 – 0.349 | 0.48 |
|  |  | VM | 0.06 | -0.199 – 0.291 | 0.68 |
|  | Stride length | IM | 0.13 | -0.136 – 0.366 | 0.33 |
|  |  | VM | 0.03 | -0.224 – 0.266 | 0.83 |
|  | CV_stride-time_ | IM | 0.06 | -0.201 – 0.308 | 0.66 |
|  |  | VM | -0.08 | -0.310 – 0.148 | 0.55 |

**Table S7. Gold-MSI descriptive statistics**

| **Gold-MSI subscale** | **Mean** | **SD** | **Median** | **Min** | **Max** |
| --- | --- | --- | --- | --- | --- |
| Active engagement | 27.0 | 12.9 | 23.5 | 9 | 56 |
| Perceptual abilities | 49.4 | 8.4 | 49.0 | 30 | 63 |
| Musical training | 14.9 | 11.3 | 7.0 | 7 | 43 |
| Singing abilities | 30.1 | 8.1 | 30.0 | 6 | 42 |
| Emotional response | 26.1 | 5.0 | 26.0 | 13 | 38 |
| **General musical sophistication** | **60.8** | **14.3** | **58.0** | **35** | **92** |
